# Proton Pump Inhibitors Inhibit Metformin Uptake by Organic Cation Transporters (OCTs)

**DOI:** 10.1371/journal.pone.0022163

**Published:** 2011-07-14

**Authors:** Anne T. Nies, Ute Hofmann, Claudia Resch, Elke Schaeffeler, Maria Rius, Matthias Schwab

**Affiliations:** 1 Dr. Margarete Fischer-Bosch Institute of Clinical Pharmacology, Stuttgart, Germany; 2 University of Tuebingen, Tuebingen, Germany; 3 Division of Epigenetics, German Cancer Research Center, Heidelberg, Germany; 4 Department of Clinical Pharmacology, Institute of Experimental and Clinical Pharmacology and Toxicology, University Hospital, Tuebingen, Germany; University of Helsinki; Finland

## Abstract

Metformin, an oral insulin-sensitizing drug, is actively transported into cells by organic cation transporters (OCT) 1, 2, and 3 (encoded by *SLC22A1*, *SLC22A2*, or *SLC22A3*), which are tissue specifically expressed at significant levels in various organs such as liver, muscle, and kidney. Because metformin does not undergo hepatic metabolism, drug-drug interaction by inhibition of OCT transporters may be important. So far, comprehensive data on the interaction of proton pump inhibitors (PPIs) with OCTs are missing although PPIs are frequently used in metformin-treated patients. Using *in silico* modeling and computational analyses, we derived pharmacophore models indicating that PPIs (i.e. omeprazole, pantoprazole, lansoprazole, rabeprazole, and tenatoprazole) are potent OCT inhibitors. We then established stably transfected cell lines expressing the human uptake transporters OCT1, OCT2, or OCT3 and tested whether these PPIs inhibit OCT-mediated metformin uptake *in vitro*. All tested PPIs significantly inhibited metformin uptake by OCT1, OCT2, and OCT3 in a concentration-dependent manner. Half-maximal inhibitory concentration values (IC_50_) were in the low micromolar range (3–36 µM) and thereby in the range of IC_50_ values of other potent OCT drug inhibitors. Finally, we tested whether the PPIs are also transported by OCTs, but did not identify PPIs as OCT substrates. In conclusion, PPIs are potent inhibitors of the OCT-mediated metformin transport *in vitro*. Further studies are needed to elucidate the clinical relevance of this drug-drug interaction with potential consequences on metformin disposition and/or efficacy.

## Introduction

Metformin (1,1-dimethylbiguanide) is an oral insulin-sensitizing agent commonly used either alone or in combination with other antihyperglycemic drugs in patients with type 2 diabetes [1]. Based on various population-based analyses, prescription of metformin in patients with type 2 diabetes increased by about 50% in European countries [2]–[5].

The glucose-lowering effect of metformin is largely attributable to inhibition of hepatic gluconeogenesis, and additionally, insulin-stimulated glucose uptake into skeletal muscle cells and adipocytes is increased by metformin [1]. Recently, it has been shown that organic cation transporters (OCTs) are crucial for the uptake of metformin [6]–[10] and these membrane transport proteins are expressed at significant levels in metformin target tissues such as liver, muscle, and adipose tissue [10]–[13]. Data from OCT1 knockout mice [6], [9] as well as from healthy volunteers carrying OCT1 variants [9], [14] clearly indicate an alteration of metformin disposition and subsequent consequences for plasma glucose levels.

Since metformin does not undergo hepatic metabolism [15], [16], drug-drug interaction by inhibition of OCT transporters might be important. Because OCT1 is expressed in human liver [10], [11], alteration of hepatic metformin uptake may be assumed, thereby resulting in poor response to metformin treatment due to reduced glucose-lowering effects. Otherwise, drug-drug interaction with OCT2, which is expressed in proximal tubule epithelial cells [17], would probably increase systemic disposition of metformin by reduced renal clearance. Recently, a strong inhibiting effect of repaglinide and rosiglitazone on OCT1-mediated metformin transport as well as of several drugs on OCT2-mediated metformin transport *in vitro* has been reported [18]–[20]. Clinically, concomitant use of the potent OCT2 inhibitors cimetidine and verapamil [21] in cisplatin-treated patients resulted in a lower risk for cisplatin-related nephrotoxicity [22] since the antitumor drug cisplatin is an OCT2 substrate [23], [24]. This clinical observation is supported by animal data, clearly demonstrating that cimetidine-related inhibition of the OCT2 transporter alters cisplatin uptake in the kidney [25], [26]. These examples suggest that OCT-mediated drug-drug interactions appear to be clinically relevant.

Hundreds of xenobiotics including drugs potentially inhibiting OCTs were tested in the past and several new inhibitors have been identified [19], [21], [27]. However, systematic data regarding the important drug class of proton pump inhibitors (PPIs) are still missing although PPIs are frequently used in metformin-treated patients with metabolic syndrome and cardiovascular diseases. Moreover, gastroesophageal reflux disease (GERD) is commonly seen in patients with type 2 diabetes [28], [29] and PPIs are the drugs of best choice in treatment of GERD [30].

The aim of the present study was to investigate systematically *in vitro* the drug-drug interaction potential of PPIs with OCTs. We first used *in silico* pharmacophore modeling to assess the inhibitory potential of PPIs. We then generated cell lines stably expressing recombinant human OCT1 (encoded by the *SLC22A1* gene), OCT2 (*SLC22A2*), or OCT3 (*SLC22A3*) to experimentally confirm the inhibitory potential of PPIs on OCT-mediated metformin transport. Finally, we assessed whether PPIs are transported by OCTs. Altogether, we identified PPIs as potent inhibitors of OCT-mediated metformin transport.

## Results

### Pharmacophore Model Generation

**Figure 1:**
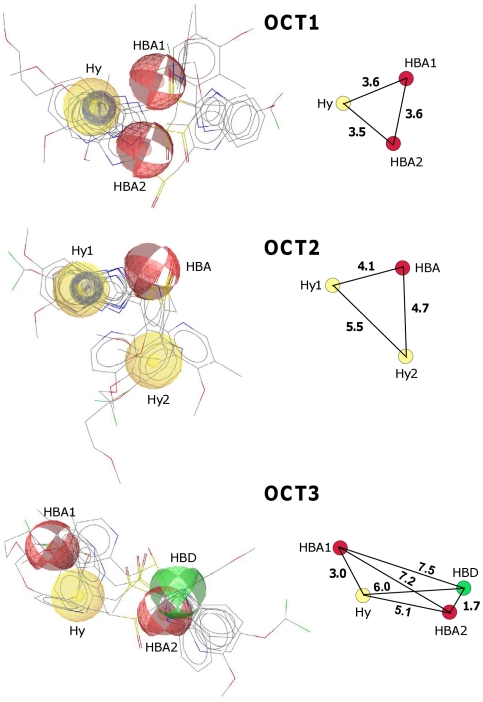
Pharmacophore models of OCT1, OCT2, and OCT3. Ligand-based pharmacophore models were generated with igandScout using the 15 most potent OCT1, OCT2, or OCT3 inhibitors as training set and 5 PPIs (omeprazole, pantoprazole, lansoprazole, rabeprazole, tenatoprazole) as test set. The 5 PPIs are mapped to each pharmacophore model. Yellow sphere: hydrophobic interaction site (Hy), red sphere: H-bond acceptor site (HBA), green sphere: H-bond donor site (HBD); 2 stacked blue rings: aromatic ring. On the right, the centers of the pharmacophore features are redrawn and the interfeature distances are shown in Ångstroms.

Pharmacophore models were constructed for all 3 OCTs. For this, the 15 most potent inhibitors of OCT1, OCT2, or OCT3 were included as training compounds to test whether the PPIs omeprazole, pantoprazole, lansoprazole, rabeprazole, and tenatoprazole are OCT inhibitors (Figure S1). This ligand-based approach revealed an OCT1 pharmacophore comprising 1 hydrophobic interaction site and 2 H-bond acceptor sites, an OCT2 model comprising 2 hydrophobic interaction sites and 1 H-bond acceptor site, and an OCT3 model comprising 1 hydrophobic interaction site, 2 H-bond acceptor sites, and 1 H-bond donor site (Figure 1, Table S1). Moreover, Figure 1 shows the relative molecular dimensions of the pharmacophore feature points. The OCT1 pharmacophore appears to have the smallest dimensions, followed by the OCT2 and the OCT3 pharmacophore.

For all 5 PPIs high pharmacophore fit values were calculated (Table S2) and each PPI occupies all the pharmacophore features that were found to be relevant for a certain pharmacophore (Figure S2). Both results suggest that the PPIs are potent OCT inhibitors.

### Characterization of OCT-expressing HEK Cells

**Figure 2:**
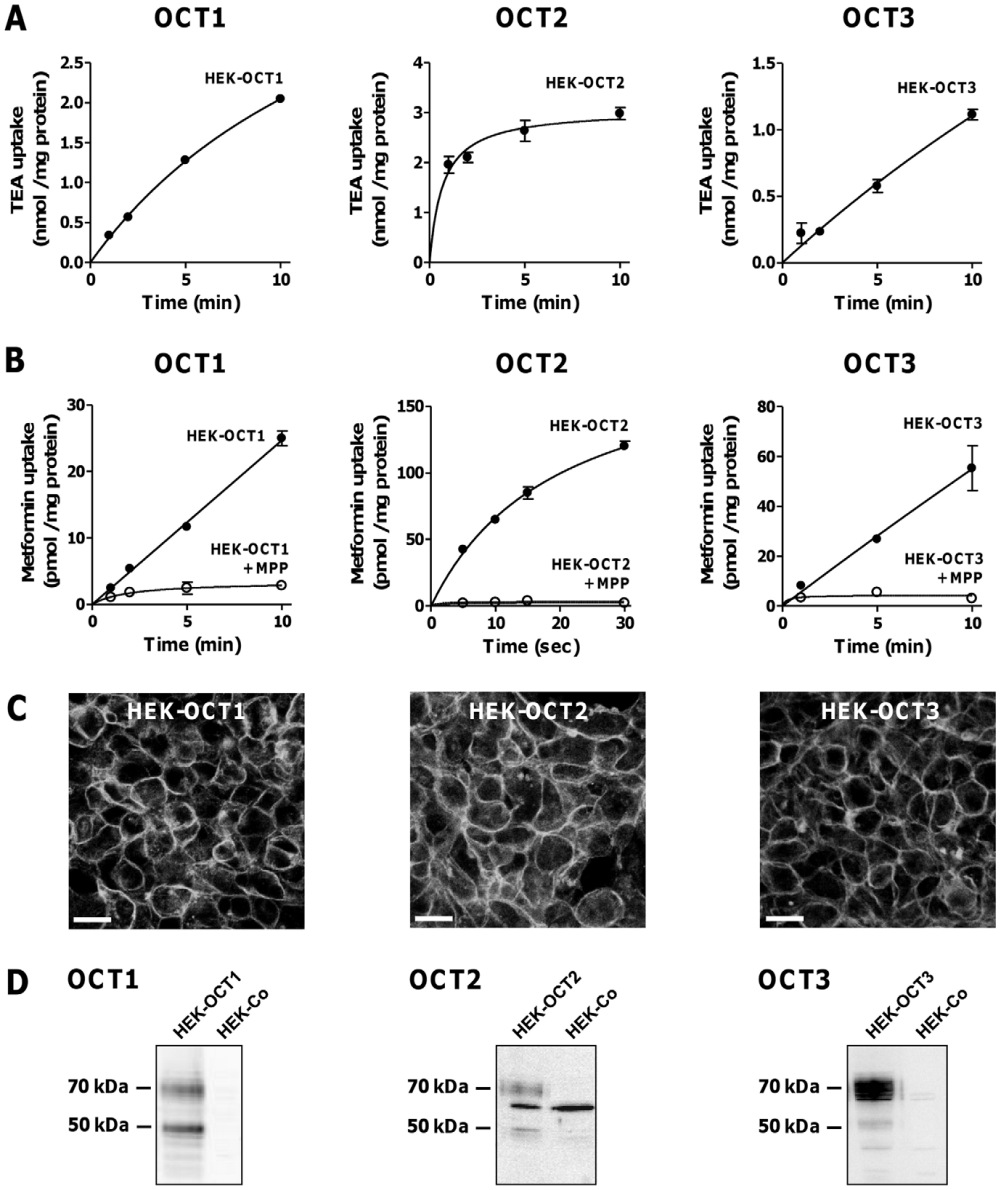
Characterization of HEK cells stably transfected with cDNAs encoding human OCT1, OCT2, or OCT3. **A.** Time-dependent uptake of 100 µM TEA, a prototypic OCT substrate, by HEK-OCT1, HEK-OCT2, or HEK-OCT3. Data are means ± SD of 3 determinations. **B.** Time-dependent uptake of 5 µM metformin by HEK-OCT1, HEK-OCT2, or HEK-OCT3 and assessment of non-specific uptake by performing uptake studies in the presence of 2 mM unlabeled MPP, a high-affinity OCT substrate. Data are means ± SD of 3 determinations. **C.** Confocal laser scanning micrographs of HEK-OCT1, HEK-OCT2, and HEK-OCT3 cells after incubation with the OCT isoform-specific antisera KEN, KEK, and CGR, respectively [10], [31]. Bars, 20 µm. **D.** Immunoblot analyses of membrane fractions from HEK-OCT1, HEK-OCT2, and HEK-OCT3 cells in comparison with those from vector-transfected control cells (HEK-Co) using the OCT isoform-specific antisera KEN, KEK, and CGR, respectively.

In order to test inhibitory potencies of PPIs *in vitro*, we stably transfected HEK cells with cDNAs encoding human OCT1, OCT2, or OCT3. All 3 transfected cell lines showed time-dependent uptake of the prototypic substrate TEA (Figure 2A) and of metformin (Figure 2B). Metformin uptake was almost completely absent in the presence of 2 mM MPP, indicating that metformin uptake is largely due to recombinantly expressed OCT transporters. As expected by these uptake experiments, the recombinant OCT proteins were detected in the plasma membrane of HEK cells by immunofluorescence microscopy (Figure 2C) using isoform-specific antibodies characterized previously [10], [31]. None of the isoform-specific antibodies resulted in staining of vector-transfected control HEK cells. The respective recombinant OCT protein was detected in membrane fractions from OCT-expressing HEK cells, but not in control cells, at molecular masses of ∼70 kDa (Figure 2D). The band at ∼50 kDa in membrane fractions from OCT1-expressing HEK cells probably reflects an isoform with a low extent of glycosylation, because after deglycosylation only one band at ∼45 kDa was detectable (not shown). The band seen at ∼65 kDa using the anti-OCT2 antibody can be most likely explained by a cross-reactivity of the antibody with a protein endogenously expressed in the HEK cells because it is detected in OCT2-expressing as well as in control HEK cells. This protein appears to be detectable only under SDS denaturing conditions since in the immunostaining experiments staining was only observed in OCT2-expressing but not in control HEK cells.

### Inhibition of OCT-mediated Metformin Transport by PPIs

**Figure 3:**
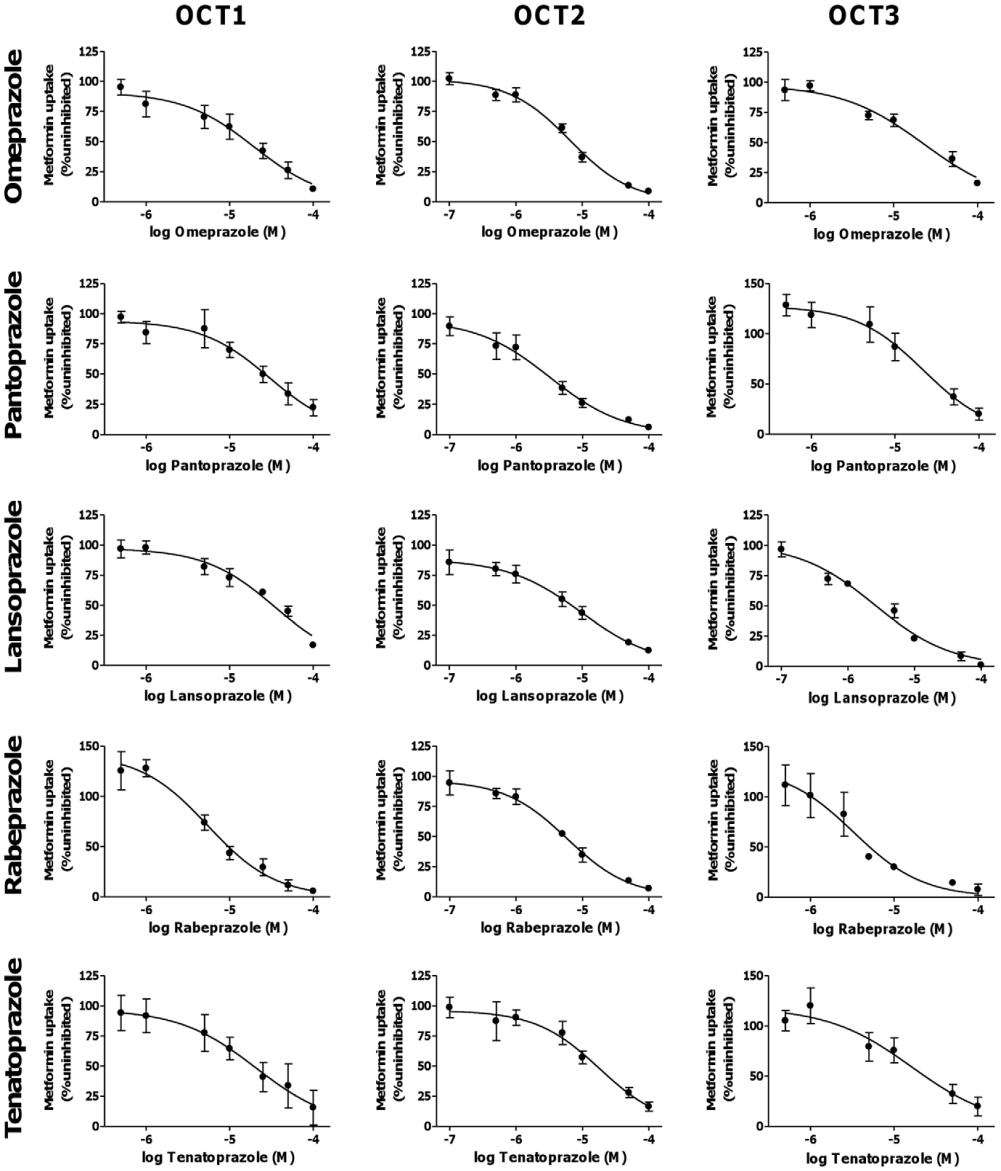
Inhibition of OCT-mediated metformin uptake by proton pump inhibitors. Uptake of 5 µM [^14^C]metformin was measured after 5 min (OCT1, OCT3) or 5 sec (OCT2) to remain within the linear uptake phase of each transporter. OCT-specific metformin uptake was obtained by subtracting metformin uptake in the presence of 2 mM unlabeled MPP from that in its absence. OCT-specific metformin uptake in the presence of different concentrations of omeprazole, pantoprazole, lansoprazole, rabeprazole, or tenatoprazole was then calculated as percentage of that in the absence of the respective PPI (100% uninhibited). Data points are the means ± SE of 3 individual experiments, with 3 parallel measurements performed in each individual experiment. Lines result from direct fits of the Hill equation to the data points.

Inhibition of OCT-specific metformin uptake by PPIs was measured within the linear uptake phase of each transporter. Each PPI significantly inhibited OCT-mediated metformin transport in a concentration-dependent manner (Figure 3). The calculated IC_50_ values are in the low micromolar range (3–36 µM; Table 1). Regarding the benzimidazoles omeprazole, pantoprazole, lansoprazole, and rabeprazole, the latter one has the highest inhibitory potency towards all 3 OCTs. Omeprazole, pantoprazole, and lansoprazole tended to inhibit OCT2 more potently than OCT1 or OCT3. For the imidazopyridine tenatoprazole, similar IC_50_ values were obtained for all 3 OCTs.

**Table 1:** Pharmacokinetic data of PPIs in humans and IC_50_ values of OCT-mediated metformin transport as determined in the present study.

PPI	Dosage(mg)	Dosage(µmol)	C_max_(µM)	Calculated C_port.vn._(µM)	IC_50_ OCT1(µM)	IC_50_ OCT2(µM)	IC_50_ OCT3(µM)
Omeprazole	20	58	1.8 (ref. [51])	5.4	15.7±3.8	6.7±2.1	22.0±2.2
Pantoprazole	40	104	5.5 (ref. [52])	12.0	30.8±8.8	2.8±0.9	22.9±5.2
Lansoprazole	30	81	2.7 (ref. [53])	7.8	35.8±5.8	9.5±3.8	3.1±1.3
Rabeprazole	20	56	1.2 (ref. [54])	4.7	3.0±1.7	5.7±0.5	3.0±0.8
Tenatoprazole	40	116	10 (ref. [55])	17.2	23.3±7.1	20.3±7.2	14.7±2.4

PPI dosage and maximal total PPI concentration in the systemic circulation (C_max_) were from the indicated references. The maximal total PPI concentration in the portal venous blood (C_port.vn_) was calculated according to equation 1 in ref. [56]. For each PPI, 3 individual experiments were performed and within an individual experiment, metformin uptake at a given PPI concentration was obtained from 3 parallel measurements. IC_50_ values were calculated for each individual experiment and are given as means ± SE.

### Relationship between Physicochemical Properties of PPIs and OCT Inhibition Potency

Because the 5 PPIs are very similar in structure, their OCT inhibition potency might be explained by a single physicochemical property. We, therefore, analyzed whether 7 computed physicochemical properties of PPIs (molecular weight, topological polar surface area [TPSA], H-bond acceptor count, rotatable bond count, tautomer count, heavy atom count, and the lipophilicity parameter “calculated log octanol/water partition coefficient” [ClogP], Table S3) correlated with the inhibitory potency towards OCTs. In univariate analyses, no significant correlation between the IC_50_ values and these physicochemical properties were observed (Table S4).

We next determined the relationship between the IC_50_ values and these 7 physicochemical parameters by partial least square (PLS) analysis. OCT1 inhibition was best explained by a two-component model. The cumulative Q^2^ value of 0.74 indicated a good predictive power of the model and the R^2^ value of 0.99 indicated that variation in the IC_50_ values was largely modeled by the two components. For comparison, a one-component model resulted in a Q^2^ value of 0.19 explaining 89% of variation in the IC_50_ values. The variable influence on projection (VIP) function was then used to identify the physicochemical parameters most relevant for explaining the IC_50_ values in the two-component model. The number of H-bond acceptor sites (VIP = 1.61) and the ClogP value (VIP = 1.15) contributed most to the two-component model for OCT1 inhibition. For OCT2 and OCT3 inhibition, the PLS analysis resulted in one-component models. Although the R^2^ values were 0.53 for OCT2 and 0.56 for OCT3, the Q^2^ values were <0 for both suggesting that the models cannot predict IC_50_ values for OCT2 and OCT3.

### Evaluation of PPIs as Transport Substrates of OCTs

**Figure 4:**
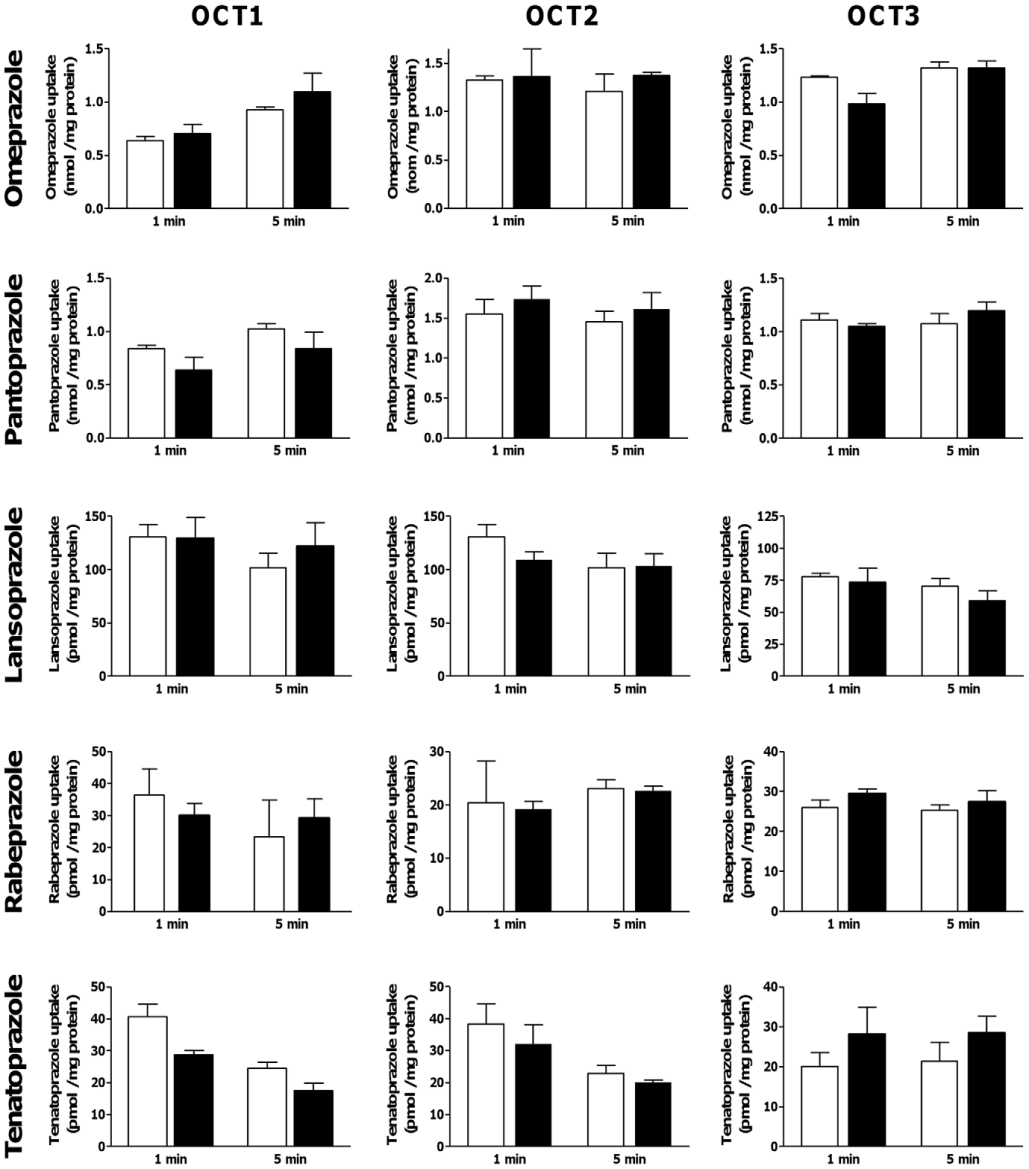
Evaluation of proton pump inhibitors as transport substrates of OCTs. Intracellular accumulation of 10 µM [^14^C]omeprazole, 10 µM [^14^C]pantoprazole, 5 µM lansoprazole, 5 µM rabeprazole, or 5 µM tenatoprazole by vector-transfected control HEK cells (white bars) or OCT-expressing HEK cells (black bars) was measured after 1 or 5 min of incubation. Data are means ± SD of 3 determinations.

In order to assess whether the 5 PPIs are transported by OCTs, PPI uptake by OCT-expressing HEK cells was compared with that by control cells after 1 and 5 min of incubation (Figure 4). None of the PPIs accumulated to higher levels in the OCT-expressing cells than in control cells indicating that PPIs are not transported by OCTs.

## Discussion

With the recent advances in the understanding of the role of drug transporters in pharmacokinetics it has become critical to elucidate drug-drug interactions that are rooted in transporters. Drug transporters can be generally classified as either uptake or efflux transporters characterizing whether they facilitate drug entry into a cell or efflux out of a cell.

In the present paper we focused on the uptake transporter proteins OCT1, OCT2, and OCT3 since the antidiabetic drug metformin is a substrate for each and there is already evidence that e.g. the antidiabetics repaglinide or rosiglitazone [18] as well as H_2_ receptor antagonists (e.g. cimetidine, [19]) inhibit OCT function. Since PPIs are frequently used in patients with type 2 diabetes, we combined *in silico* pharmacophore modeling with subsequent *in vitro* assays to systematically investigate drug-drug interaction of metformin with omeprazole, pantoprazole, lansoprazole, rabeprazole, which are FDA-approved agents, and the non-FDA-labeled PPI tenatoprazole (benatoprazole, TU-199).

The pharmacophore models described for OCT1 [32]–[34] and OCT2 [19], [35] share a hydrophobic interaction site and a positive ionizable site. The pharmacophore models of the present study are in line with these models in having at least 1 hydrophobic interaction site as well (Figure 1). The lack of a positive ionizable site in our models is probably due to the fact that many of the compounds selected for the training sets [19], [27], [36] are neutral at pH 7.4. Our pharmacophore models predict PPIs to be very potent inhibitors of OCT1, OCT2, and OCT3 (Table S1), mainly due to their hydrophobic features and presence of H-bond acceptor sites.

In order to validate the data of the in *silico* pharmacophore modeling, we generated cell systems stably expressing recombinant human OCT1, OCT2, or OCT3. All 3 transfected HEK cell lines expressed functionally active organic cation transporters as demonstrated by time-dependent TEA and metformin uptake (Figure 2A and B), which are both well-established substrates of OCTs (reviewed in [21]). Consistent with these functional data, the recombinant OCT proteins were detected in the plasma membrane of the OCT-expressing HEK cells (Figure 2C) as well as in membrane fractions from these cells (Figure 2D) as expected [10], [31].

The most striking result of our study was a potent inhibition of metformin uptake transport by all five PPIs for all 3 OCT proteins tested (OCT1, OCT2, and OCT3) with IC_50_ values in the low micromolar range, similar to calculated total PPI concentrations in portal venous blood (Figure 3, Table 1). Moreover, we could clearly show that none of these PPIs are substrates for the 3 OCT transport proteins (Figure 4). The fact that drugs are potent OCT inhibitors without being substrates, is in agreement with results obtained for several other compounds (reviewed in ref. [21]).

Moreover, OCT1- and OCT3-mediated metformin uptake appears to be activated by low concentrations of selected PPIs (OCT1: by rabeprazole; OCT3: by tenatoprazole, pantoprazole, rabeprazole; Figure 3), which is in line with previous observations reported for carvedilol and OCT2-mediated metformin uptake [37] but also for other uptake transporters (e.g. OATP1B3) and inhibitors (e.g. rosiglitazone) [18]. However, underlying molecular mechanisms are currently unknown.

Given the role of OCT1 for metformin action [9] and of OCT2 for renal secretion of metformin [8], efforts have been made to identify physicochemical parameters that may predict whether a compound inhibits the OCT transporters. One study showed that a positive charge at pH 7.4 and a high lipophilicity (ClogP ∼3.5) are the main properties of potent OCT1 inhibitors [27]. The PLS analysis revealed that the ClogP value likewise appears to be a relevant factor for explaining OCT1 inhibition by the 5 PPIs. For OCT2, one study also identified the ClogP value as a principal factor for potent inhibition [35], while in another study the TPSA value was predictive for inhibition [19]. However, neither the ClogP value nor the TPSA value are apparently predictive for OCT2 or OCT3 inhibition by PPIs. It therefore remains unclear which physicochemical parameters determine the inhibition potency of PPIs towards OCT2 and OCT3. Another physicochemical parameter, i.e. the charge at pH 7.4 that was identified as a relevant property of OCT1 inhibitors [27], is apparently not sufficient for predicting a compound's inhibition potency towards OCTs since PPIs are neutral at pH 7.4 and it has been shown that several other OCT inhibitors are likewise not positively charged (e.g. prazosin, amsacrine, phenoxybenzamine, lamivudine, azidothymidine).

Currently, to the best of our knowledge no interaction studies in healthy volunteers and/or patients exist elucidating pharmacokinetic and/or –dynamic consequences of a combined therapy of metformin and PPIs. Although it is difficult to predict clinical consequences based on *in vitro* data there are some issues supporting such an assumption.

Since OCT1 and OCT3 are expressed in the plasma membrane of human hepatocytes, skeletal muscle cells, and adipocytes [10], [12], [13], [31], an inhibition potential of OCT function by PPIs may abolish the glucose-lowering effect of metformin. This assumption is corroborated by the observation that OCT1/3-mediated metformin uptake into murine hepatocytes [9], human adipocytes [13] or human skeletal muscle cells [12] is significantly reduced by known OCT inhibitors such as quinidine or cimetidine. Similarly, the activating effect of metformin on AMP-activated protein kinase is altered [9], [12], [13].

Moreover, certain genotypes may directly affect the inhibition potential of a drug, a mechanism which is increasingly recognized [38]. OCT1 pharmacogenetics and drug-drug interaction for metformin uptake and selected OCT1 inhibitors (e.g. verapamil or amitriptylin) have recently been reported by *in vitro* experiments [39]. Interestingly, an increased sensitivity to drug inhibition was observed for OCT variants, particularly for those with reduced function. While the glucose-lowering effect of metformin is impaired either in healthy volunteers [9] or in diabetic patients [40] carrying OCT1 variants with reduced function [14], no data are currently available with respect to PPI co-medication. Since generally only 60% of metformin-treated patients with type 2 diabetes do respond well [41], it might be possible that, clinically, PPIs are a yet unrecognized factor for insufficient metformin response due to a transporter-mediated drug-drug interaction via OCT transport proteins.

Taken together, we identified PPIs as an important drug class inhibiting OCT-mediated metformin transport. Moreover, our present work underscores the impact of in *silico* pharmacophore modeling since these computational data have been confirmed by our experimental studies using transfected cell lines, which express functionally active OCTs. Additionally, this newly recognized *in vitro* drug-drug interaction warrants further clinical studies to elucidate the *in vivo* relevance in metformin-treated patients regarding drug disposition and/or pharmacodynamic consequences.

## Materials and Methods

### Pharmacophore Model Generation

Pharmacophore models for OCT1, OCT2, and OCT3 were generated with LigandScout (version 3.01, Inte:Ligand, Vienna, Austria; ref. [42], [43]) using the ligand-based strategy. With this strategy, the pharmacophore model is derived from a set of ligands in the absence of a macromolecule structure. This approach searches for a common feature pattern that is shared in an active ligand set and considers the conformational flexibility of the ligands. All LigandScout parameters were kept default and models were generated with the Fast calculation setting and a maximal number of 75 conformations. For the OCT1 pharmacophore, the training set consisted of the 10 most potent inhibitors as based on IC_50_ values (5–17 µM) reported by Ahlin et al. [27]. Moreover, the nucleotide reverse transcriptase inhibitors (NRTIs) lamivudine, abacavir, tenofovir, azidothymidine, and emtricitabine were included in the training set since they were recently identified to interact with OCTs with high-affinity (IC_50_ values in the low nanomolar range; [44]). The OCT2 pharmacophore model was constructed with the 10 most potent inhibitors described by Zolk et al. [19] (IC_50_ values from 6–25 µM) and the NRTIs as training compounds. Because for OCT3 no comprehensive inhibition analysis is available, compounds with the lowest IC_50_ values (0.8–20 µM) were included from several studies (compiled from ref. [21]), as well as the NRTIs. The PPIs omeprazole, pantoprazole, lansoprazole, rabeprazole, and tenatoprazole were included for each pharmacophore model generation as test compounds. Cartesian coordinates and tolerance radii of chemical features of the pharmacophore models were exported in xyz file format. Cartesian coordinates were then imported into PyMOL (version 1.3, Schroedinger LLC, Portland, OR), which was used to measure interfeature distances.

### Chemicals and Antibodies

[^14^C]Tetraethyl ammonium (TEA, MBq⋅mmol^−1^) and [^14^C]metformin hydrochloride (4074 MBq⋅mmol^−1^) were from PerkinElmer (Rodgau, Germany) and Hartmann Analytic (Braunschweig, Germany), respectively. [^14^C]Omeprazole (470 MBq⋅mmol^−1^) and [^14^C]pantoprazole (550 MBq⋅mmol^−1^) were generously supplied by Byk Gulden (Konstanz, Germany) as described [45]. Pantoprazole, rabeprazole, and tenatoprazole were from Sequoia Research Products Ltd. (Pangbourne, UK). All other chemicals, including G418 (geneticin), hygromycin, omeprazole, and lansoprazole were from Sigma-Aldrich (Taufkirchen, Germany) or Merck (Darmstadt, Germany) at the highest grade available. Generation and use of the isoform-specific polyclonal antisera directed against human OCT1 (KEN), OCT2 (KEK), or OCT3 (CGR) have been described previously [10], [31].

### Cloning of Human OCTs and Generation of Stably Transfected HEK Cell Lines

*SLC22A1* cDNA and *SLC22A2* cDNA had been PCR-amplified from human liver and kidney, respectively, as reported elsewhere and cDNAs were provided by the German Cancer Research Center [31]; the study and the use of the tissue were approved by the institutional review board of the German Cancer Center, Heidelberg, Germany. Since the data were analyzed anonymously, and tissues were left-over samples from diagnostic procedures, written informed consent had not been required and obtained. IRB specifically waived the need for consent due to the fact the human tissue material used was fully anonymized. *SLC22A3* cDNA was amplified using SLC22A3-TrueClone (Origene, Rockville, MD, USA) as the template. *SLC22A1* cDNA was cloned into the expression vector pcDNA3.1/Hygro(-) (Invitrogen, Darmstadt, Germany), *SLC22A2* and *SLC22A3* cDNA into pcDNA3.1(+) (Invitrogen). Each transporter cDNA was sequenced and the respective deduced amino acid sequence was identical to the published reference protein sequence (OCT1: NP_003048.1; OCT2: NP_003049.2; OCT3: NP_068812.1). The human embryonic kidney 293 (HEK) cell line was obtained from the American Type Culture Collection. HEK cells were grown in DMEM (Sigma) supplemented with 10% fetal bovine serum (Sigma), 100 U⋅ml^−1^ penicillin, and 100 µg⋅ml^−1^ streptomycin (Lonza, Basel, Switzerland) at 37°C and 5% CO_2_. HEK cells were transfected with Metafectene Pro (Biontex, München, Germany). Selection of clones stably expressing OCT1 was carried out with 0.5 mg⋅ml^−1^ hygromycin as described [31]. Similarly, clones stably expressing OCT2 or OCT3 were selected with 800 µg⋅ml^−1^ G418. HEK cells stably transfected with the empty pcDNA3.1(+) vector served as controls (HEK-Co). Cells were incubated with 5 mM butyrate 24 h before use to increase protein levels of recombinant transporters.

### Immunolocalization Analyses

Transfected HEK cells were grown for 2 days on glass slides and fixed with methanol at −20°C for 10 min. Cells were then incubated with the primary antibodies and subsequently the Alexa488-conjugated goat anti-rabbit secondary antibody (1∶300, Invitrogen/Molecular Probes) for 1 h as described [10]. Primary antibodies were diluted in PBS as follows: KEN antiserum (against OCT1) 1∶300, KEK antiserum (against OCT2) 1∶100, CGR antiserum (against OCT3) 1∶100. Images were taken with a confocal laser scanning microscope (TCS NT Confocal System, Leica Microsystems, Wetzlar, Germany).

### Immunoblot Analyses

Membrane fractions were prepared from transfected HEK cells as described [10] and stored at −80°C. Prior to immunoblotting, membrane fractions were denatured for 30 min at 37°C in Laemmli sample buffer and separated on 10% SDS/polyacrylamide gels. Immunoblotting was performed essentially as described [10]. Primary antibodies were incubated with the nitrocellulose membranes for 1 h at room temperature at the following dilutions: KEN antiserum 1∶3000, KEK antiserum 1∶2000, CGR antiserum 1∶3000. Membranes were developed with enhanced chemoluminescence detection solution (Supersignal WestDura, Pierce, Rockford, IL, USA) and chemoluminescence was measured with a charged-coupled device camera (Fuji LAS-1000, Raytest, Straubenhardt, Germany). In some cases, membrane fractions were deglycosylated with peptide *N*-glycosidase F (EC 3.5.1.52) according to the manufacturer's instructions (New England BioLabs, Ipswich, MA) and then subjected to gel electrophoresis.

### Metformin and PPI Transport Studies

HEK cells (400000 cells/well) were seeded into 24-well cell culture plates (Greiner Bio-One, Frickenhausen, Germany) and grown for 48 h. Cell adherence was improved by coating plates with poly-L-lysine (Biochrom AG, Berlin, Germany) prior to cell plating. All uptake studies were carried out at 37°C as described [31]. For uptake measurements, cells were firstly washed with uptake buffer (145 mM NaCl, 5 mM hydroxyethylpiperazine ethanesulfonic acid, 3 mM KCl, 1 mM CaCl_2_, 0.5 mM MgCl_2_, 5 mM glucose, pH 7.4, [46]) prewarmed to 37°C. Uptake was initiated by replacing this solution with uptake buffer containing 5 µM [^14^C]metformin, 100 µM [^14^C]TEA, 10 µM [^14^C]omeprazole, or 10 µM [^14^C]pantoprazole. Uptake was stopped at indicated time points by removing the uptake buffer and immediately washing the cells 3 times with ice-cold uptake buffer. Cells were lysed with 0.2% SDS and intracellular radioactivity was determined by liquid scintillation counting (Hidex 300SL TDCR liquid scintillation counter, Turku, Finland). For measuring inhibition of OCT-mediated metformin uptake by PPIs, 5 µM [^14^C]metformin uptake was measured in the presence of different PPI concentrations and stopped after 5 min (OCT1, OCT3) or 5 sec (OCT2).

For determination of intracellular accumulation of unlabeled lansoprazole, rabeprazole, and tenatoprazole, cells were incubated for 1 or 5 min with 5 µM PPI at 37°C and then immediately washed three times with ice-cold uptake buffer and twice with ice-cold phosphate-buffered saline. Cells were harvested by scraping them off in 500 µl 0.1 M sodium carbonate buffer (pH 9.3) : methanol 4∶1 (v/v) and then transferred into Eppendorf tubes. Cells were disrupted by three cycles of shock freezing/thawing (liquid nitrogen, 37°C water bath) and further by ultra-sonification, three times 3 sec, at 4°C. Disrupted cell solutions were centrifuged 5 min (15000 g) at 4°C and supernatants were transferred into Eppendorf tubes and stored at −20°C for analytic determination of the PPIs.

Protein content of lysed cells was determined in 25-µl aliquots using the bicinchoninic acid assay as described [31].

### Determination of Lansoprazole, Rabeprazole, and Tenatoprazole

Lansoprazole, rabeprazole, and tenatoprazole were determined by LC-MS using omeprazole as internal standard similar to a method described for omeprazole [47]. An Agilent Series 1100 LC-MSD system (Agilent, Waldbronn, Germany) with binary pump, degasser, autosampler and mass selective detector equipped with an electrospray ion source was used. Chromatographic separation was achieved on a ProntoSil AQ, C18 column (3 mm i.d. x 150 mm, particle size 3 µm, Bischoff, Leonberg, Germany) at a flow rate of 0.5 ml⋅min^−1^. Gradient runs with the mobile phases (A) 10 mM ammonium acetate in water and (B) acetonitrile were programmed as described previously [47]. Electrospray parameters were as follows: capillary voltage 4000 V, drying gas flow 9.5 l⋅min^−1^ nitrogen, drying gas temperature 350°C, and nebulizer pressure 30 psig (207 kPa gauge). The mass spectrometer was operated in the selected ion monitoring mode using the respective MH^+^ ions, *m/z* 370 for lansoprazole, *m/z* 360 for rabeprazole, *m/z* 347 for tenatoprazole, and *m/z* 346 for the internal standard omeprazole. The fragmentor was set at 80 V for lansoprazole, rabeprazole and omeprazole, and at 60 V for tenatoprazole.

Calibration samples were prepared in 0.1 M sodium carbonate buffer pH 9.3 : methanol 4∶1 (v/v) containing 1 µM omeprazole as internal standard, in the concentration range 2.5 to 250 nM for lansoprazole and rabeprazole, and from 5 to 250 nM for tenatoprazole. Calibration samples were worked up as the samples, and analyzed together with the unknown samples. Calibration curves based on internal standard calibration were obtained by weighted (1/x^2^) linear regression for the peak area ratio of the analyte to the internal standard against the amount of the analyte. The concentration in unknown samples was obtained from the regression line.

### Calculation of IC_50_ Values and Correlation with Physicochemical Properties

OCT-specific metformin uptake was obtained by subtracting metformin uptake in the presence of 2 mM unlabeled 1-methyl-4-phenylpyridinium (MPP) from that in its absence. MPP was used because it is transported by all 3 OCTs with high-affinity (K_m_ of OCT1: 15–32 µM; K_m_ of OCT2: 3–19 µM; K_m_ of OCT3: 47–83 µM; reviewed in ref. [21]). OCT-specific metformin uptake in the presence of a PPI was then calculated as percentage of that in the absence of the respective PPI (100% uninhibited). For each PPI concentration, 3 individual experiments each with 3 parallel measurements were performed. Apparent IC_50_ values for inhibition of metformin uptake by PPIs were calculated for each individual experiment by fitting the values to the Hill equation using GraphPad Prism 4 (GraphPad Software Inc., La Jolla, CA, USA). Values in Table 1 are given as the means ± SE of the 3 individual experiments.

The 2 physicochemical parameters “calculated octanol/water partition coefficient” (ClogP) and “charge at pH 7.4” were calculated using MarvinView (version 5.4.1.1, ChemAxon Ltd., Budapest, Hungary). Seven other computed physicochemical properties (molecular weight, topological polar surface area [TPSA], H-bond donor count, H-bond acceptor count, rotatable bond count, tautomer count, heavy atom count) were from the publicly available PubChem Compound database (http://www.ncbi.nlm.nih.gov/pccompound; ref. [48]). TPSA estimation and H-bond donor/acceptor classification follow refs. [49] and [50], respectively.

For the analysis of the relationship between physicochemical properties of PPIs and IC_50_ values, the 7 parameters molecular weight, ClogP, TPSA, H-bond acceptor count, rotatable bond count, tautomer count, and heavy atom count were included, because the 2 parameters “charge at pH 7.4” and “H-bond donor count” did not vary among the PPIs. For univariate analysis, the Pearson correlation coefficient (R) and P values (two-tailed) were calculated with GraphPad Prism 4. Normality of data was confirmed by the Kolmogorov-Smirnov test. Statistical significance was defined as P<0.05. Multivariate analyses were performed with the Excel-based add-in tool XLSTAT (version 2011.2.05; Addinsoft, Paris, France) using the partial least square analysis module. The 7 physicochemical parameters molecular weight, ClogP, TPSA, H-bond acceptor count, rotatable bond count, tautomer count, and heavy atom count were included as quantitative descriptive x values and the IC_50_ values as quantitative dependent y values. The R^2^ value indicates how much of variation of the IC_50_ values is explained by the components. The quality of the fit is estimated by the Q^2^ value. A Q^2^>0 indicates that the model has a better predictive power than no model.

## Supporting Information

Figure S1 Molecular structures of the 5 tested PPIs.
(TIF)

Figure S2 Structural formula of the 5 PPIs with those chemical features marked that were found by LigandScout to be relevant for the OCT1, OCT2, or OCT3 pharmacophore model.
(TIF)

Table S1 Chemical feature coordinates and tolerance radii (in Ångstroms) found by LigandScout for the pharmacophore models of OCT1, OCT2, and OCT3.
(DOC)

Table S2 Pharmacophore fit values of training sets and PPIs as test set.
(DOC)

Table S3 Physicochemical properties of the tested PPIs.
(DOC)

Table S4 Relationship of physicochemical properties of the tested PPIs and IC_50_ values of OCT1, OCT2, and OCT3 as determined by univariate analysis.
(DOC)
