# Supplementary figures and images for: Proton Pump Inhibitors Inhibit Metformin Uptake by Organic Cation Transporters (OCTs)
Source: PLoS One. 2011 Jul 14;6(7):e22163. doi: 10.1371/journal.pone.0022163 (PMC3136501; doi:10.1371/journal.pone.0022163)

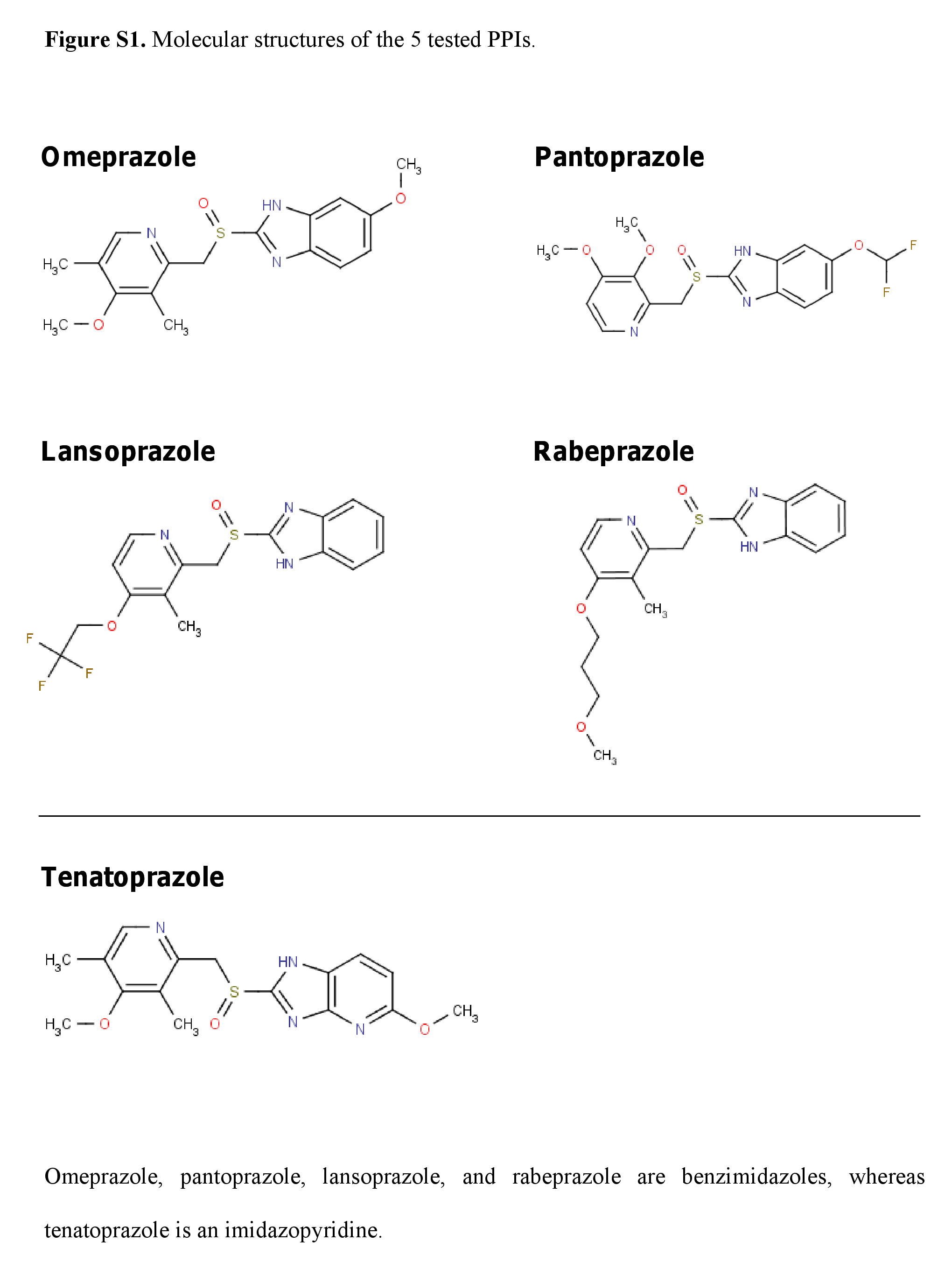

Supplement: Figure S1 — Molecular structures of the 5 tested PPIs. (TIF) [file pone.0022163.s001.tif]

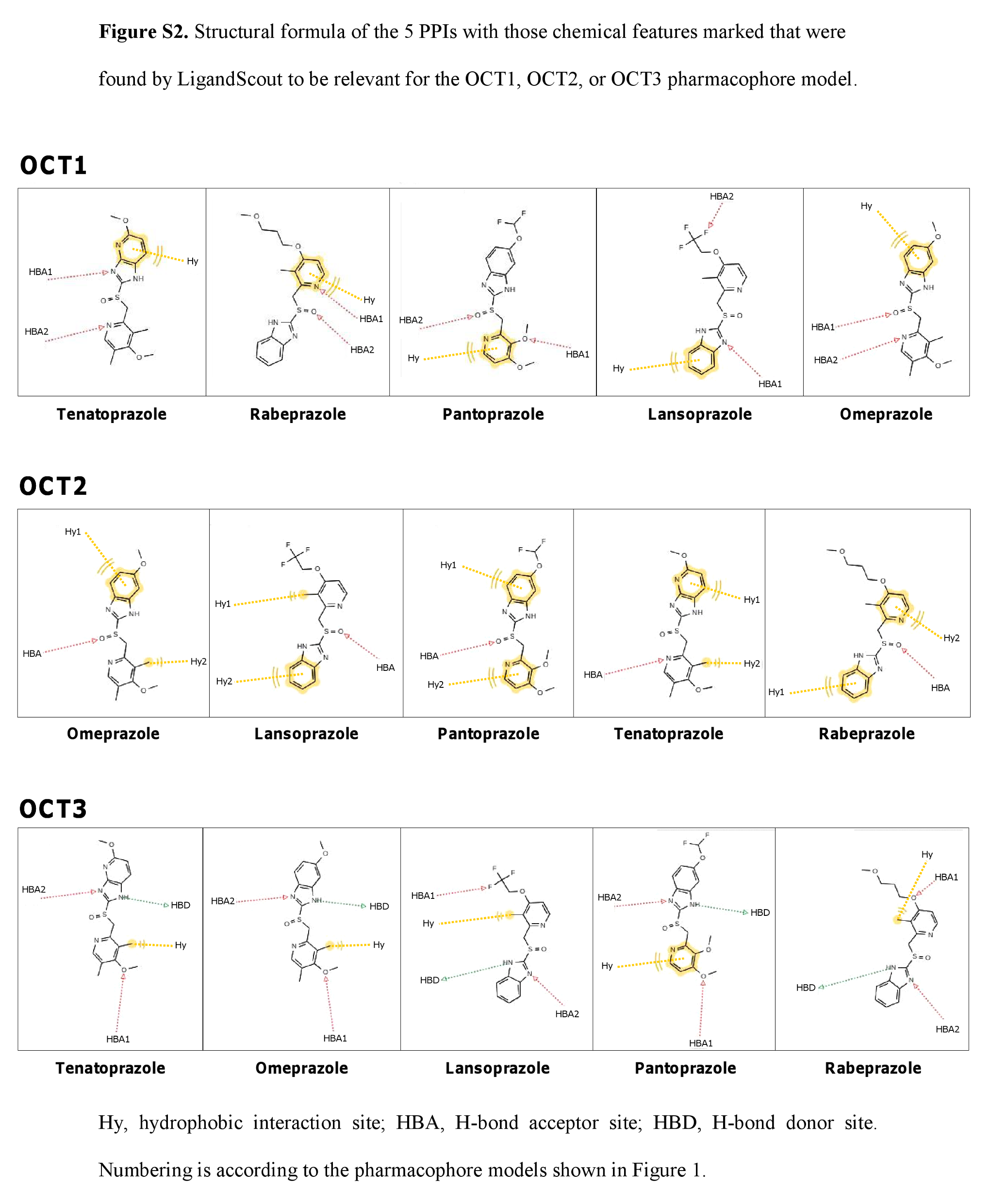

Supplement: Figure S2 — Structural formula of the 5 PPIs with those chemical features marked that were found by LigandScout to be relevant for the OCT1, OCT2, or OCT3 pharmacophore model. (TIF) [file pone.0022163.s002.tif]
