# Supplementary material for: Proton Pump Inhibitors Inhibit Metformin Uptake by Organic Cation Transporters (OCTs)
Source: PLoS One. 2011 Jul 14;6(7):e22163. doi: 10.1371/journal.pone.0022163 (PMC3136501; doi:10.1371/journal.pone.0022163)
Supplement: Table S1 — Chemical feature coordinates and tolerance radii (in Ångstroms) found by LigandScout for the pharmacophore models of OCT1, OCT2, and OCT3. (DOC) [file pone.0022163.s003.doc]

**Table S1.** Chemical feature coordinates and tolerance radii (in Ångstroms) found by LigandScout for the pharmacophore models of OCT1, OCT2, and OCT3.

**OCT1**

| **Feature type** | **Radius** | **Cartesian coordinates**  **X Y Z** | | |
| --- | --- | --- | --- | --- |
| Aromatic ring | 0.9 | 3.00 | 1.77 | -0.16 |
| Hydrophobic site | 1.5 | 2.95 | 2.14 | -0.18 |
| H-bond acceptor 1 | 1.5 | 4.19 | -0.52 | 1.94 |
| H-bond acceptor 2 | 1.5 | 2.88 | -1.16 | -1.32 |

**OCT2**

| **Feature type** | **Radius** | **Cartesian coordinates**  **X Y Z** | | |
| --- | --- | --- | --- | --- |
| Aromatic ring | 0.9 | 5.50 | -2.98 | 3.38 |
| Hydrophobic site 1 | 1.5 | 5.72 | -3.17 | 3.17 |
| Hydrophobic site 2 | 1.5 | 1.06 | -1.03 | 1.17 |
| H-bond acceptor | 1.5 | 3.57 | -0.24 | 5.01 |

**OCT3**

| **Feature type** | **Radius** | **Cartesian coordinates**  **X Y Z** | | |
| --- | --- | --- | --- | --- |
| Hydrophobic site | 1.5 | 0.35 | 0.22 | -1.63 |
| H-bond acceptor 1 | 1.5 | -2.88 | -1.17 | -5.33 |
| H-bond acceptor 2 | 1.5 | -0.53 | 1.24 | 1.06 |
| H-bond donor | 1.5 | -4.49 | -1.31 | -4.74 |
