# Supplementary material for: Proton Pump Inhibitors Inhibit Metformin Uptake by Organic Cation Transporters (OCTs)
Source: PLoS One. 2011 Jul 14;6(7):e22163. doi: 10.1371/journal.pone.0022163 (PMC3136501; doi:10.1371/journal.pone.0022163)
Supplement: Table S2 — Pharmacophore fit values of training sets and PPIs as test set. (DOC) [file pone.0022163.s004.doc]

**Table S2.** Pharmacophore fit values of training sets and PPIs as test set.

OCT1

| **Compound** | **Type** | **Pharmacophore fit value** | **Charge at pH 7.4** |
| --- | --- | --- | --- |
| Propafenone | Training | 41.5 | +1 |
| Repaglinide | Training | 41.4 | -1 |
| Verapamil | Training | 41.3 | +1 |
| Lamivudine | Training | 41.2 | 0 |
| Pantoprazole | Test / PPI | 40.6 | 0 |
| Tenatoprazole | Test / PPI | 40.2 | 0 |
| Diltiazem | Training | 40.1 | +1 |
| Atropine | Training | 40.1 | +1 |
| Lansoprazole | Test / PPI | 40.0 | 0 |
| Rabeprazole | Test / PPI | 39.5 | 0 |
| Amsacrine | Training | 39.2 | 0 |
| Omeprazole | Test / PPI | 38.8 | 0 |
| Prazosin | Training | 34.1 | 0 |
| Tenofovir | Training | 32.9 | -1.4 |
| Clemastine | Training | 32.8 | +1 |
| Azidothymidine | Training | 32.2 | 0 |
| Emtricitabine | Training | 31.6 | 0 |
| Abacavir | Training | 31.4 | +0.1 |
| Phenoxybenzamine | Training | 25.7 | 0 |
| Amitriptyline | Training | 25.6 | +1 |

OCT2

| **Compound** | **Type** | **Pharmacophore fit value** | **Charge at pH 7.4** |
| --- | --- | --- | --- |
| Propafenone | Training | 40.0 | +1 |
| Diphenhydramine | Training | 39.5 | +1 |
| Chlorpromazine | Training | 38.5 | +1 |
| Doxepin | Training | 38.2 | +1 |
| Tenatoprazole | Test / PPI | 38.1 | 0 |
| Pantoprazole | Test / PPI | 38.0 | 0 |
| Omeprazole | Test / PPI | 37.9 | 0 |
| Lansoprazole | Test / PPI | 37.8 | 0 |
| Rabeprazole | Test / PPI | 37.6 | 0 |
| Fenfluramine | Training | 37.6 | +1 |
| Abacavir | Training | 31.8 | +0.1 |
| Clonidine | Training | 31.7 | +1 |
| Imipramine | Training | 31.6 | +1 |
| Tenofovir | Training | 31.3 | -1.4 |
| Ipratropium | Training | 31.1 | 0 |
| Sibutramine | Training | 30.6 | +1 |
| Amitriptyline | Training | 30.6 | +1 |
| Lamivudine | Training | 23.0 | 0 |
| Azidothymidine | Training | 23.0 | 0 |
| Emtricitabine | Training | 21.6 | 0 |

OCT3

| **Compound** | **Type** | **Pharmacophore fit value** | **Charge at pH 7.4** |
| --- | --- | --- | --- |
| Lansoprazole | Test / PPI | 40.7 | 0 |
| Pantoprazole | Test / PPI | 40.4 | 0 |
| Tenatoprazole | Test / PPI | 40.3 | 0 |
| Omeprazole | Test / PPI | 40.3 | 0 |
| Rabeprazole | Test / PPI | 40.1 | 0 |
| Pentamidine | Training | 39.7 | +2 |
| Abacavir | Training | 39.3 | +0.1 |
| Quinidine | Training | 39.3 | +1 |
| Tenofovir | Training | 39.2 | -1.4 |
| Lamivudine | Training | 38.7 | 0 |
| Vincristine | Training | 38.7 | +1.8 |
| Emtricitabine | Training | 38.3 | 0 |
| Azidothymidine | Training | 32.7 | 0 |
| Famotidine | Training | 32.7 | +1 |
| Phenytoin | Training | 32.6 | 0 |
| Prazosin | Training | 32.0 | 0 |
| Furamidine | Training | 31.3 | +2 |
| Irinotecan | Training | 31.2 | +1 |
| Desipramine | Training | 23.5 | +1 |
| Phenoxybenzamine | Training | 14.8 | 0 |

Pharmacophore models were generated with LigandScout using the 15 most potent OCT1, OCT2, or OCT3 inhibitors as training set and the 5 PPIs as test set. Charge at pH 7.4 was calculated with MarvinView.
