# Supplementary material for: Proton Pump Inhibitors Inhibit Metformin Uptake by Organic Cation Transporters (OCTs)
Source: PLoS One. 2011 Jul 14;6(7):e22163. doi: 10.1371/journal.pone.0022163 (PMC3136501; doi:10.1371/journal.pone.0022163)
Supplement: Table S3 — Physicochemical properties of the tested PPIs. (DOC) [file pone.0022163.s005.doc]

**Table S3.** Physicochemical properties of the tested PPIs.

|  | Omeprazole | Pantoprazole | Lansoprazole | Rabeprazole | Tenatoprazole |
| --- | --- | --- | --- | --- | --- |
| CID | 4594 | 4679 | 3883 | 5029 | 636411 |
| Molecular weight | 345.4 | 383.4 | 369.4 | 359.4 | 346.4 |
| ClogP | 2.43 | 2.18 | 3.03 | 2.09 | 2.14 |
| Charge at pH 7.4 | 0 | 0 | 0 | 0 | 0 |
| TPSA | 96.3 | 106 | 87.1 | 96.3 | 109 |
| H-bond donors | 1 | 1 | 1 | 1 | 1 |
| H-bond acceptors | 5 | 8 | 7 | 5 | 6 |
| Rotatable bond count | 5 | 7 | 5 | 8 | 5 |
| Tautomer count | 2 | 2 | 0 | 0 | 3 |
| Heavy atom count | 24 | 26 | 25 | 25 | 24 |

CID, PubChem compound ID (http://www.ncbi.nlm.nih.gov/pccompound); ClogP, calculated log octanol/water partition coefficient; TPSA, topological polar surface area
