# Supplementary material for: Proton Pump Inhibitors Inhibit Metformin Uptake by Organic Cation Transporters (OCTs)
Source: PLoS One. 2011 Jul 14;6(7):e22163. doi: 10.1371/journal.pone.0022163 (PMC3136501; doi:10.1371/journal.pone.0022163)
Supplement: Table S4 — Relationship of physicochemical properties of the tested PPIs and IC50 values of OCT1, OCT2, and OCT3 as determined by univariate analysis. (DOC) [file pone.0022163.s006.doc]

**Table S4.** Relationship of physicochemical properties of the tested PPIs and IC_50_ values of OCT1, OCT2, and OCT3 as determined by univariate analysis.

|  | OCT1 | OCT2 | OCT3 |
| --- | --- | --- | --- |
| Molecular weight | 0.508 (0.38) | -0.596 (0.29) | -0.017 (0.98) |
| ClogP | 0.600 (0.29) | -0.029 (0.96) | -0.360 (0.55) |
| TPSA | -0.047 (0.94) | 0.334 (0.58) | 0.608 (0.28) |
| H-bond acceptor count | 0.844 (0.07) | -0.179 (0.77) | 0.212 (0.73) |
| Rotatable bond count | -0.519 (0.37) | -0.584 (0.30) | -0.198 (0.75) |
| Tautomer count | 0.156 (0.80) | 0.466 (0.43) | 0.802 (0.10) |
| Heavy atom count | 0.312 (0.61) | -0.672 (0.21) | -0.020 (0.97) |

Values are Pearson R correlation coefficients and P values (in brackets). ClogP, calculated log octanol/water partition coefficient; TPSA, topological polar surface area
